# In Situ Gel-Forming System for the Removal of Ferruginous Deposits on Nanhai I Shipwreck

**DOI:** 10.3390/gels11070543

**Published:** 2025-07-12

**Authors:** Jianrui Zha, Ruyi Wang, Jing Du, Naisheng Li, Xiangna Han

**Affiliations:** 1 Key Laboratory of Archaeomaterials and Conservation, Ministry of Education, Institute of Cultural Heritage and History of Science and Technology, University of Science and Technology Beijing, Beijing 100083, China; jrzha@ustb.edu.cn (J.Z.); ustbwangruyi@163.com (R.W.); jayna422@ustb.edu.cn (X.H.); 2 National Centre for Archaeology, 21A Zhuanjiaolounanli, Chaoyang District, Beijing 100013, China

**Keywords:** wooden components, ferruginous deposits cleaning, carboxymethyl chitosan/tannic acid, in situ gel forming, Nanhai I Shipwreck

## Abstract

The removal of iron deposits on shipwreck surfaces by mechanical cleaning is labour-intensive work. This study develops an in situ gel and peeling cleaning method, utilising a carboxymethyl chitosan/tannic acid (CMCS/TA) colloidal solution spray on the surface of ferruginous deposits, promoting their removal by adhesion, chelation, and electrostatic bonding processes. The investigation confirmed that the CMTA-2 sample exhibited a sprayable viscosity of 263 mPa/s, the largest single removal thickness of 1.01 mm, a significant reduction in the fe/s atomic ratio by 2.53 units, and enhanced the deposit removal homogeneity. The field testing of the Nanhai I cultural relic showed a 14.37% reduction in iron concentration and a significant decrease in red colour (Δa* = 4.36). The synergistic mechanism involves TA chelating Fe^2+^/Fe^3+^ ions, while the CMCS gel network facilitates interfacial adhesion and mechanical peeling, hence promoting efficient and controllable cleaning.

## 1. Introduction

Nanhai I, a merchant vessel from the Southern Song Dynasty (1127–1279 AD), was rescued in 2007 and is currently preserved in the Guangdong Maritime Silk Road Museum, providing critical insights into historic shipbuilding and maritime trade [1,2,3]. Over 800 years of ocean immersion resulted in iron deposits on the surface of artefacts [4,5]. The energy spectrum analysis of the wood from the Nanhai I shipwreck reveals a significant sulphur content, whereas the XRD analysis indicates that the ferruginous deposits in the wood predominantly consist of pyrite (FeS_2_), rhodochrosite (FeCO_3_), hydroxyl ferric oxide (FeOOH), magnetite (Fe_3_O_4_), and iron sulphate (Fe_2_(SO_4_)_3_) [6,7,8,9].

The ferruginous deposits negatively impact the shipwreck in three ways. Firstly, the marine burial environment is oxygen deficient, which restricts microbial activity. The reduced sulphur–iron compounds are highly susceptible to oxidation and the generation of sulfuric acid in the presence of water. This exacerbates the degradation of cellulose in wood. Secondly, Fe^2+^/Fe^3+^ will serve as catalytic function in the oxidation of ferruginous deposits and the degradation of organic matter through the Fenton reaction. The oxidative degradation of wood generates organic acids, including formic acid, ethanoic acid, and oxalic acid, which will further damage the hull structure. In addition, the gradual oxidation of ferruginous deposits results in volumetric expansion, causing stress-induced damage [1,10,11,12,13]. In summary, the ferruginous deposits damage wooden shipwreck structures, risking the long-term stability of the Nanhai I shipwreck. Therefore, the removal of ferruginous deposits from the wooden hull is necessary.

Current removal methods including chemical, electrophoretic, biological, supercritical fluid, and vapour-phase techniques, which exhibit considerable limitations. Arc’Antique have shown that electrophoresis facilitates the extraction of Fe^2+^ ions from artefacts by directing their migration towards the cathode. Nevertheless, its efficacy is restricted to tiny or slender items owing to diffusion constraints [14]. The biological method efficiently removes iron/sulphur species with no residues detected by Raman spectroscopy. However, its efficacy is wood species-dependent, thus limiting its application [15]. Yishu et al. used Acidithiobacillus ferrooxidans to efficiently extract iron from marine waterlogged wood (Nanhai I), as confirmed by ICP-AES and conductivity tests. FTIR measurements revealed progressive wood degradation (↑I_1505_/I_1365_ ratio) due to acidification [16]. Céline et al. used CO_2_ supercritical fluid extraction to effectively remove iron/sulphur compounds (89.6% elimination) while minimizing wood damage due to near-room-temperature operation. Nonetheless, high equipment costs and reduced efficacy for polar organosulfur compounds without modifiers restricted the application [17]. Fors et al.’s ammonia vapour treatment (Batavia) effectively removed deposits (2.85 kg from 169 kg wood). The requirement for a 48 h airtight exposure during vapour treatment complicates large-scale operations and requires post-treatment ventilation [18]. The four aforementioned methods impose strict requirements for handling the environment and equipment, and the cleaning efficiency for large immovable ships (Nanhai I) is comparatively inadequate.

Chemical cleaning enables high-efficiency and safe iron deposit removal. Zhang et al. used EDTAHO to extracted 303.60 mg/L of Fe from Qing Dynasty shipwrecks in Ningbo after 12 days, achieving a rate 156% faster than EDTA-2Na under near-neutral conditions (ΔpH = 0.63), therefore preventing acid degradation, while preserving wood integrity for long-term conservation [19,20,21]. Gunnar et al. immersed wood samples from Vasa in a mixed solution of EDDHMA/NaOH, as EDDHMA possesses the ability to capture iron ions, facilitating the solubility of sulphur–iron compounds [18]. Zhang et al. evaluated EDTA-2Na, diethylenetriamine pentaacetic acid (DTPA) [22], tribasic ammonium citrate (C_6_H_17_N_3_O_7_), sodium oxalate (Na_2_C_2_O_4_), amidoxime cellulose, deferoxamine mesylate (DFO) [23], and mixtures with H_2_O_2_ or Na_2_S_2_O_4_. Amidoxime cellulose and neutral DTPA (pH 7.6) showed the highest sustained iron removal efficacy over 365 days, with amidoxime cellulose being a promising material. DTPA-H_2_O_2_ initially boosted short-term removal but subsequently decreased, whereas EDTA-2Na-Na_2_S_2_O_4_ significantly enhanced the long-term performance of EDTA. Citrate and oxalate formed iron oxide precipitates at pH levels exceeding 7, reducing efficacy. DFO exhibited subpar performance levels despite significant theoretical stability. Alkaline DTPA (pH 9.0) proved less effective than neutral DTPA due to the precipitation of FeOOH. Chemical cleaning effectively chelates Fe^2+^/Fe^3+^ in neutral/mildly acidic conditions, though issues in controllability remain.

To evaluate the cleaning influence, researchers assess the alterations in wood dimensions, the degree of wood degradation, the variation in ferruginous deposit content, and colour variance. Elisa et al. confirmed that EDTA-4Na disintegrates hemicellulose, resulting in partial mass loss, increased viscous behaviour, and modifications to mechanical integrity. Neutral or acidic agents (EDTA-2Na/citric acid) preserve wood properties with minimal impact [24].

Regarding the shortcomings of chemical cleaning methods, the gel cleaning method offers advantages, such as an environmentally friendly regulated cleaning area. Gels can be mixed with an appropriate complexing agent, oxidizing agent, buffer, etc., and can exhibit high selectivity and effectively adsorb deposits and rust [25]. Inspired by this, we propose a cleaning gel that, when sprayed onto the surfaces of cultural relics in situ, the gel process forms a colloidal film that dries and strips away ferruginous deposits, thereby streamlining the cleaning process and improve efficiency. Since the Nanhai I shipwreck has undergone PEG reinforcement, the cleaning agent must contain elements capable of remove PEG and ferruginous deposits.

CMCS is derived from the natural polysaccharide chitosan. Due to its good biocompatibility, biodegradability and capacity for metal complexation, CMCS has been widely used in the fields of environmental protection, drug delivery, and the conservation of cultural relics [26]. Li et al. introduced polyelectrolyte poly (sulfobetaine methacrylate to methylacryloyloxyethyl trimethylammonium) (PSD) into a poly vinyl alcohol (PVA)/CMCS gel system. The process enhanced intermolecular electrostatic interactions, modified the porous structure and dissolution behaviour, thereby enhancing mechanical properties, and enabled the hydrogel to adsorb metal ions during mural cleaning [27]. The inclusion of -NH_2_ and -COOH groups in CMCS gels makes CMCS positively charged due to mesoamino acid protonation under acidic conditions, hence increasing its susceptibility to solubilization and the removal of PEGs at low pH levels [28].

TA is derived from plants and is prevalent in nature. It is characterized by several active phenolic hydroxyl groups in its structure, which have the capacity to form a five-membered cyclic chelate with iron ions in a reticular configuration [29,30]. TA molecules, characterised by a five-polyphenol-arm structure [31], may interact with polymer chains through hydrogen or ionic interactions and facilitate cross-linking through coordinate bonds in the presence of Fe ions [32]. ÜÇER et al. proposed that the adsorption mechanism of Fe ions and tannins involves ion exchange, complex formation, and surface adsorption [33]. Zhang et al. designed a TA-CS (chitosan)-PEG coacervate powder absorbs interfacial water to form adhesive gels with inherent antimicrobial/antioxidant functionality. Such systems offer scalable production and immediate on-site application [34].

Given the immovable nature of the Nanhai I shipwreck, we hypothesise that a CMCS/TA system will achieve the safe and effective removal of composite deposits through synergistic mechanisms. CMCS utilises protonated amino groups in acidic conditions to dissolve polyethylene glycol (PEG) reinforcements, while TA’s polyphenolic structure chelates iron deposits via Fe^3+^ coordination. Simultaneously, hydrogen bonding between CMCS amino groups and TA phenolic hydroxyls facilitates in situ cross-linking into a stabilised three-dimensional network during air-drying, thereby eliminating ferruginous deposits and PEG residues while preserving the structural integrity of waterlogged archaeological wood.

## 2. Results and Discussion

In this study, the concentration of CMCS was used to regulate the reaction between TA and to control the cleaning efficiency of ferruginous deposits. Following the cleaning process involving mixed colloidal solutions, a gel film containing iron was obtained. Samples of one-component CMCS and TA cleaning solutions were also prepared for performance comparison. In this paper, the mixed colloidal solution samples were designated CMTA-1/2/3 based on the concentration gradient.

### 2.1. Cleaning Solution Characterisation

#### 2.1.1. Viscosity Characterisation

**Figure 1:**
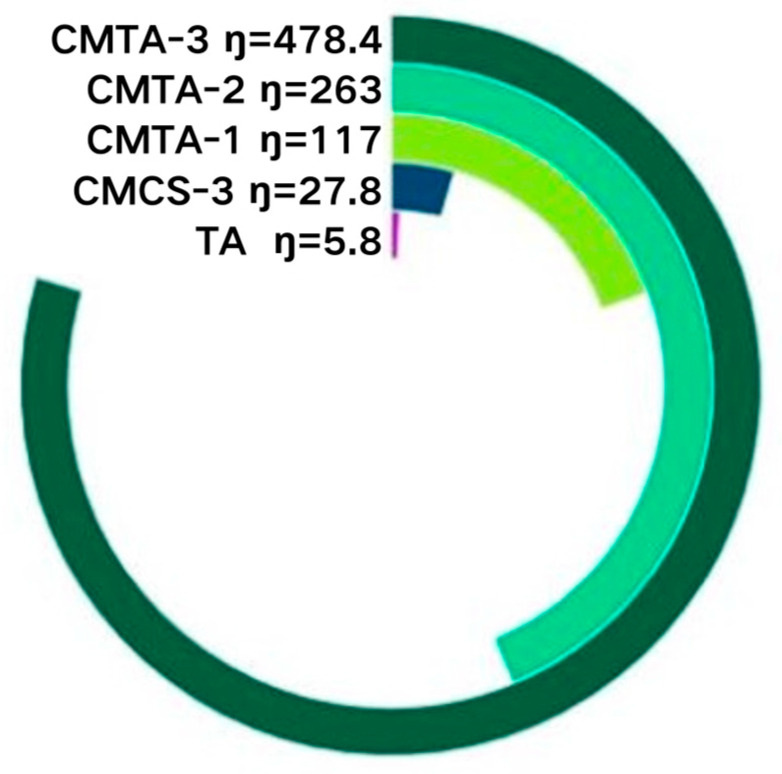
Viscosity value of cleaning solution.

Viscosity influences the spray ability of the cleaning solution, excessive viscosity may result in nozzle clogging or spraying challenges, while insufficient viscosity can cause inconsistent spraying, penetration, and fluting. The viscosity of the cleaning solution was initially assessed. Figure 1 illustrates the viscosity values of different sample solutions, revealing that the viscosity escalated with the rising carboxymethyl chitosan concentration. Specifically, the TA viscosity was the smallest at 5.3 mPa/s, followed by the CMCS viscosity at 27.8 mPa/s, and as the CMTA-1/2/3 concentration increased from 2% to 4% and then to 6%, the viscosity rose from 117 mPa/s to 263 mPa/s and then to 478.4 mPa/s. This viscosity surge directly indicates progressive gel network formation. The tests performed using the spray bottle indicated that liquids with a viscosity below 30 mPa/s exhibited mobility and were easier to spray, while those with viscosities under 300 mPa/s demonstrated fitting fluidity, making them capable of spraying. Carboxymethyl chitosan is a linear polymer polysaccharide, and its solution viscosity mostly arises from the physical entanglement of molecular chains and intramolecular/intermolecular interactions. Therefore, as the quantity of the additive rises, the density of polymer chains per unit volume of solution markedly increases, thereby enhancing the degree of molecular chains entanglement and resulting in a denser network structure. Collectively, this manifests as enhanced flow resistance (viscosity rise)—a signature of incipient gelation.

#### 2.1.2. FT-IR Characterisation

**Figure 2:**
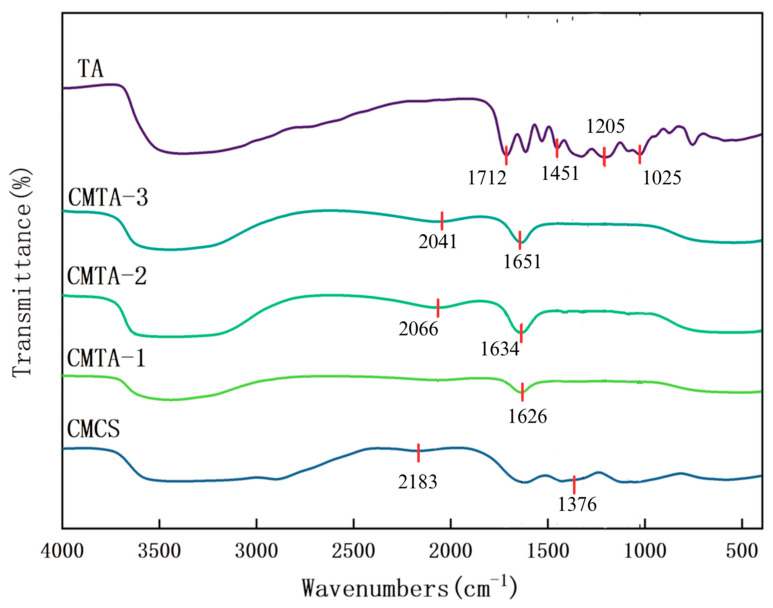
FT-IR spectra of raw materials and cleaning solutions.

FT-IR measurements are used to identify changes in the functional groups of cleaning solutions. Figure 2 displays the FT-IR spectra of raw materials and cleaning solutions. All CMTA formulations shows characteristic peaks near 1634 cm^−1^ (CMTA-1 at 1626 cm^−1^ and CMTA-3 at 1651 cm^−1^), corresponding to the asymmetric stretching vibration of COO. The characteristic peak of CMCS at 1376 cm^−1^ (N-H bending of -NH_2_) disappears in all CMTA formulations, confirming hydrogen bond formation between TA phenolic hydroxyls and CMCS amino groups. As the concentration of CMCS increases, additional functional groups do not emerge, but the COO^−^ asymmetric stretching vibration peak shifts progressively from 1626 cm^−1^ (CMTA-1) to 1634 cm^−1^ (CMTA-2) and 1651 cm^−1^ (CMTA-3), indicating an increase in hydrogen bonding strength between TA and CMCS. The weak peaks observed at 2183 cm^−1^ (CMCS) and 2066 cm^−1^ (CMTA-2) are likely attributable to interactions between the amino groups (–NH_2_) of CMCS. These peaks are absent in CMTA-1 and TA compositions with a high proportion of TA. The distinct peak of TA (raw material) shows the peak at 1712 cm^−1^, 1451 cm^−1^, 1205 cm^−1^, and 1025 cm^−1^ in pure TA corresponding to the ester group (C=O) stretching vibration, C-H bending, phenolic C-O stretch, and C-O-C glycosidic bond, and the disappearance of peak of tannic acid may be due to the hydrogen bond shielding effect between tannic acid and carboxymethyl chitosan. This interaction leads to the expansion or shift in the C=O vibrational peak to the low-frequency region. The vibrations of COO- that appeared after mixing may result from the formation of multiple hydrogen bonds between the carboxylate (-COO-) of CMCS and the phenolic hydroxyl group of TA, leading to the splitting of the asymmetric and symmetric vibration peaks and the occurrence of ionic cross-linking. The presence of hydrogen bonding and a cross-linking structure after the solution is converted into a thin film preserves the mechanical strength and facilitates deposit removal [32,35].

### 2.2. Application Effects Characterisation

#### 2.2.1. Three-Dimensional Super Depth-of-Field Microscope (3D-SDF)

**Figure 3:**
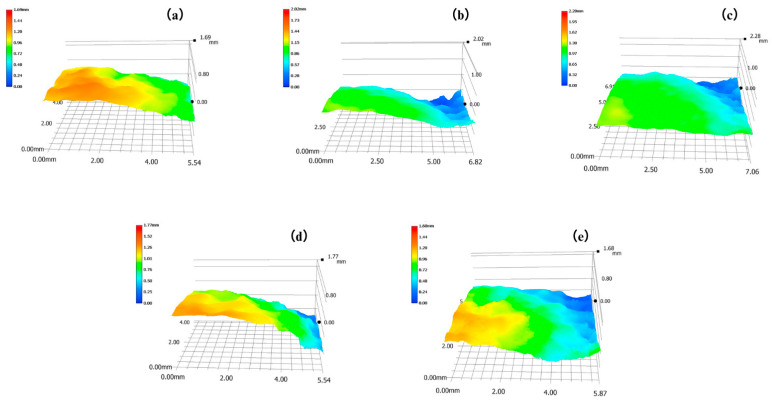
Microscope images of (**a**) CMCS, (**b**) CMTA-1, (**c**) CMTA-2, (**d**) CMTA-3, and (**e**) TA.

To assess the cleaning efficacy, the removed ferruginous deposits and substrate were measured. Figure 3 displays the 3D Super Depth-of-Field Microscope analysis results of the sample. Table 1 shows the single-removal thickness and roughness change before and after cleaning. The CMCS, CMTA-1, CMTA-2, CMTA-3, and TA thicknesses removed by a single cleaning were 0.49 mm, 1.02 mm, 1.01 mm, 0.82 mm, and 0.79 mm, respectively. The corresponding changes in surface roughness after a single clean were +0.02 mm, +0.15 mm, +0.37 mm, +0.17 mm, and −0.20 mm for CMCS, CMTA-1, CMTA-2, CMTA-3, and TA, respectively. A distinct boundary was observed between the cleaned and uncleaned areas on all samples. CMTA composites chelate deposits and peel off during film formation, increasing the cleaned-area roughness. TA dissolves iron oxide via soluble complexes, reducing roughness. CMTA-1/2/3 leverage TA’s chelation for rapid iron sulphide dissolution while CMCS gel stabilises the interface, enabling more complete reactions. Thus, CMTA removal thicknesses exceed TA/CMCS alone. Optimal TA:CMCS ratios (1:2–1:4) and moderate viscosity (CMTA-1/2) maximise efficiency. CMTA-2’s significant roughness increase (+0.37 mm) indicates strong gel–sediment adhesion, favouring artefact cleaning via gel-mediated interfacial behaviour [36].

#### 2.2.2. SEM-EDS

**Figure 4:**
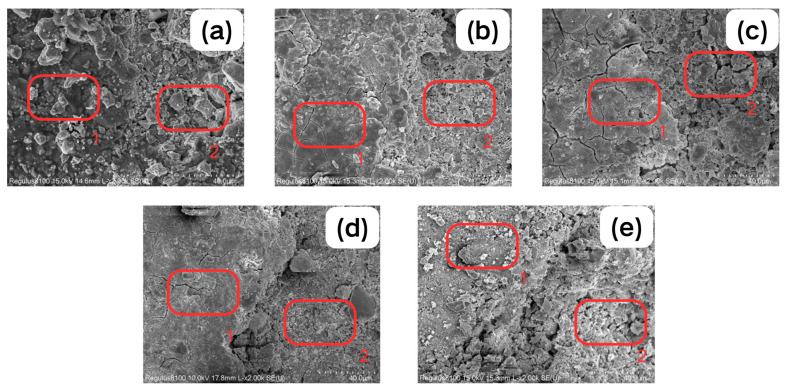
SEM images of (**a**) CMCS, (**b**) CMTA-1, (**c**) CMTA-2, (**d**) CMTA-3, and (**e**) TA.

To investigate the cleaning process, an SEM-EDS analysis was conducted at the boundary of the removed area and substrates. Figure 4 illustrates the cleaned substrate, where significant changes can be observed in the clean areas of all CMTA formulations (1–3): the surface structure became loose, but the concentration difference was not particularly significant in the substrate structure changes. Due to the mechanical separation caused by physical effects, the particles in Figure 4a are more disordered. In contrast, Figure 4c shows the longest crack length, while Figure 4b,d show narrower cracks that have formed, indicating that the gel penetration is limited under this formula. Figure 4e mainly shows the dissolution of sediment by TA solution, so there is a significant difference from the previous CMTA formulations stable chelation through interface interaction.

The EDS analysis results of both sides is specified in Figure 4 and Table 2. EDS analyses demonstrated a significant reduction in the Fe/S atomic ratio within the cleaned zones following treatment with CMTA-1 and CMTA-2 composite cleaners. This phenomenon arises from the disproportionate increase in sulphur (S) compared to iron (Fe) in the cleaned areas. These results suggest that the gel preferentially remove iron-rich phases, leading to relative sulphide enrichment in the residue, potentially via sulphide transformations. Conversely, Figure 4a,d,e exhibit increased Fe/S atomic ratios in their cleaned zones. This trend is attributed to iron accumulation exceeding sulphur enrichment, confirming that the gel cleaning process primarily exposed iron-rich deposit layers, while physical stripping (CMCS/CMTA-3 gel films) or weak chemical dissolution (TA) failed to disrupt deep iron–sulphur bonding structures. Notably, CMTA-2 achieved the largest Fe/S atomic ratio reduction (Δ = −2.53) and the lowest post-cleaning ratio (2.96). Combined with previous results, this is evidence of the enhanced dissociation of Fe-S bonds through CMTA-2’s optimised gel network, enabling deeper deposit removal and establishing it as the prime gel-mediated cleaning candidate for artefacts.

#### 2.2.3. Mapping

**Figure 5:**
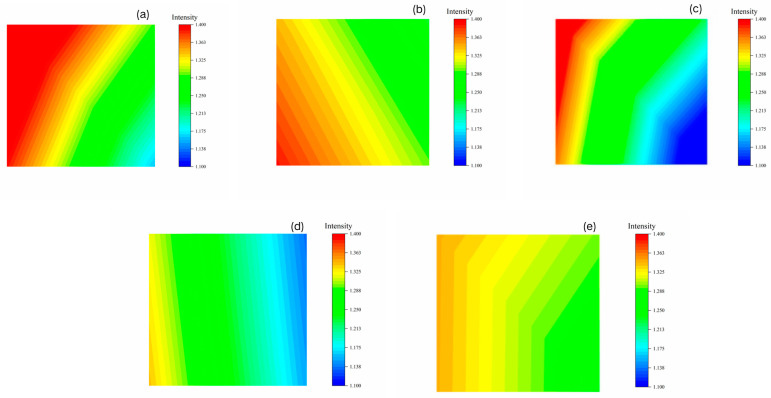
Microinfrared maps of (**a**) CMCS, (**b**) CMTA-1, (**c**) CMTA-2, (**d**) CMTA-3, and (**e**) TA after cleaning.

Microinfrared maps (Mapping) revealed alterations in the substrate surface. The characteristic peak is identified at 850 cm^−1^, corresponding to the vibration in the Fe-O bond. The change in the distinctive peaks on both sides can be visualised by the colour gradient (e.g., red highlighting signifies high intensity) in the surface scan picture. The left side of Figure 5 depicts the uncleaned area, whereas the right side illustrates the cleaned area, and the absorption intensity of Fe-O bond significantly diminished after cleaning. TA has strong liquid mobility and poor controllability, which also has some influence on the intensity of the absorption peaks in the uncleaned area. Table 3 shows the percentage of CMCS, CMTA-1, CMTA-2, CMTA-3, and TA in the high-intensity region (1.325–1.400) (51.41%, 43.56%, 19.90%, 3.01%, 28.21%), medium-intensity region (1.175–1.325) (48.19%, 56.44%, 60.11%, 79.86%, 71.79%), and low-intensity region (1.100–1.175) (0.4%, 0%, 19.99%, 17.13%, 0%). The percentages of CMCS, TA, and CMTA-1 in the high-intensity region are greater than 20%, suggesting that the cleaning efficiency is not as good as that of CMTA-2/3, and may achieve a shallow cleaning depth due to an excessive gel network [37].

#### 2.2.4. SEM

**Figure 6:**
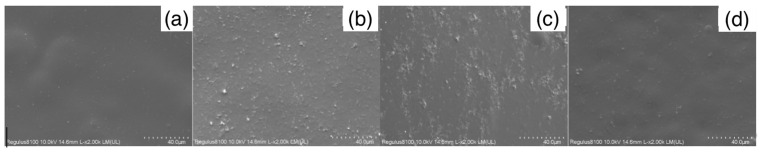
SEM images of peeled gel film for (**a**) CMCS, (**b**) CMTA-1, (**c**) CMTA-2, and (**d**) CMTA-3.

Figure 6 shows the peeled film of CMCS-, CMTA-1-, CMTA-2-, and CMTA-3-treated samples, and TA was unable to form a film. The results indicate the peeled film contains several particles, though no obvious holes were detected. The SEM image of gel film formed after natural drying without cleaning is relatively complete and smooth overall, with good film-forming properties (Supplementary Figure S1). The surface of CMTA-2 contains more particles than CMTA-1 and CMTA-3, and CMTA-2 presents a clear mesh structure. This result shows that the film has a certain mechanical strength.

#### 2.2.5. FT-IR

**Figure 7:**
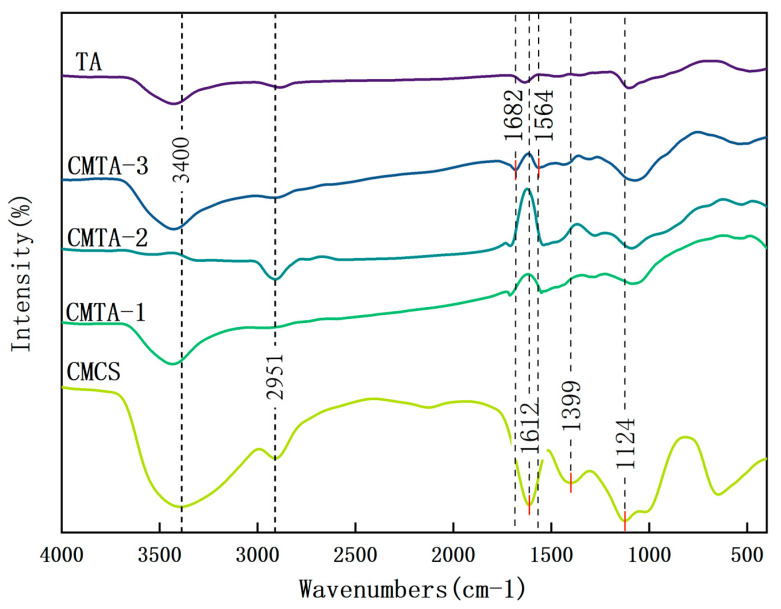
FT-IR spectra of peeled gel film.

The FT-IR results of the cleaned area are shown in Figure 7, where all samples exhibited a distinctive peak near 1124 cm^−1^, which can be ascribed to C-O-C glycosidic bond vibrations or residual sulphate groups (SO_4_^2−^) from ferruginous deposits. The CMTA sample exhibited prominent peaks at 1682 cm^−1^ (amide I band) and 1564 cm^−1^ (asymmetric COO^−^ stretching), with the latter’s shift suggesting hydrogen bonding or carboxylate-Fe^3+^ coordination. Notably, CMTA-2 exhibited a pronounced peak at 2951 cm^−1^, indicative of aliphatic C-H stretching vibrations, which reflect structure changes in the composite matrix. In contrast, the CMCS sample retained a COO^−^ peak at 1399 cm^−1^, implying weaker metal coordination than CMTA. These results demonstrate that the peeled gel film effectively removed the deposit.

#### 2.2.6. FTIR Mapping

**Figure 8:**
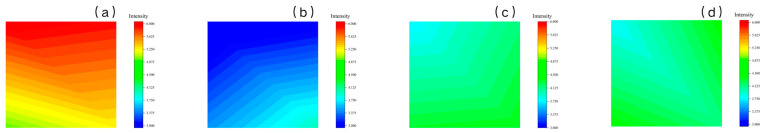
FT-IR mapping result of peeled gel film for (**a**) CMCS, (**b**) CMTA-1, (**c**) CMTA-2, and (**d**) CMTA-3.

FTIR mapping was employed to quantify residual ferruginous deposits and assess the cleaning performance. Figure 8 presents the results of infrared out-scan analysis of the film surface after cleaning, where the characteristic peak of ferruginous deposits on the cleaned film appeared at 750 cm^−1^. A comparative analysis of different film showed that the characteristic peak distribution on the surfaces of CMTA-2 and CMTA-3 was more uniform. The deposits on the CMCS film surface were predominantly bound in particulate form, leading to significant fluctuations and instability in characteristic peak intensity. Moreover, the distinctive peak intensities of CMTA-2 and CMTA-3 were in the range 3.750~4.875, exhibiting a relatively balanced distribution. These results fully demonstrate that CMTA-2 and CMTA-3 can achieve the uniform removal of ferruginous deposits, highlighting their excellent cleaning homogeneity and reliability.

### 2.3. Cleaning Mechanism

**Figure 9:**
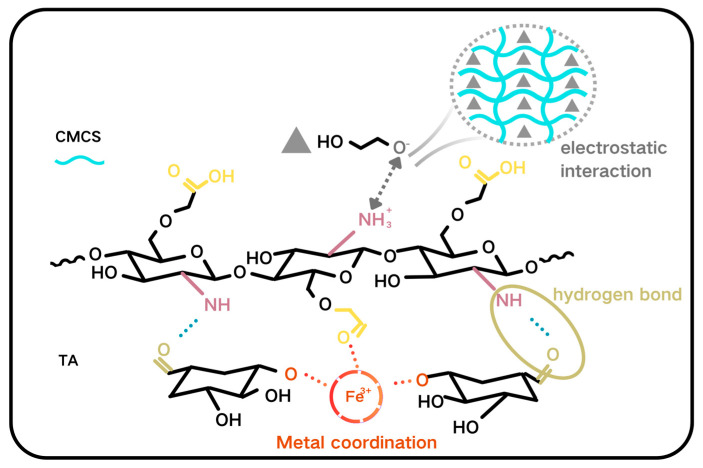
Cleaning mechanism of colloidal solutions.

Figure 9 shows the cleaning mechanism. When TA is compounded with carboxymethyl chitosan (CMCS), the formation of hydrogen bonding and the ionic cross-linking structure results in a significant improvement of the structure stability and strength of the gel film. During the cleaning process, the colloidal solution can closely fit the deposit surface and penetrate into the micro fissures. The gel film can effectively remove the deposits during stripping and enhance the cleaning efficiency. TA coordinates with Fe^2+^/Fe^3+^ ions to form a stable complex, which promotes the leaching of iron from the artefact surface and removes it with the film formation. The coordination of carboxylate root of CMCS with Fe^3+^ effectively integrates the gel film with the samples, hence enhancing the rate of coordination reaction. The ferruginous deposits removed by physical adhesion and chemical dissolutions. The oxygen atom (-O-) in the PEG ether bond carries a partially negative charge, and under acidic conditions, it electrostatically attracts the -NH^3+^ of CMCS. PEG molecular chains can penetrate the CMCS grid and be physically entangled [32,38].

### 2.4. Field Application Experiment

#### 2.4.1. XRF

**Figure 10:**
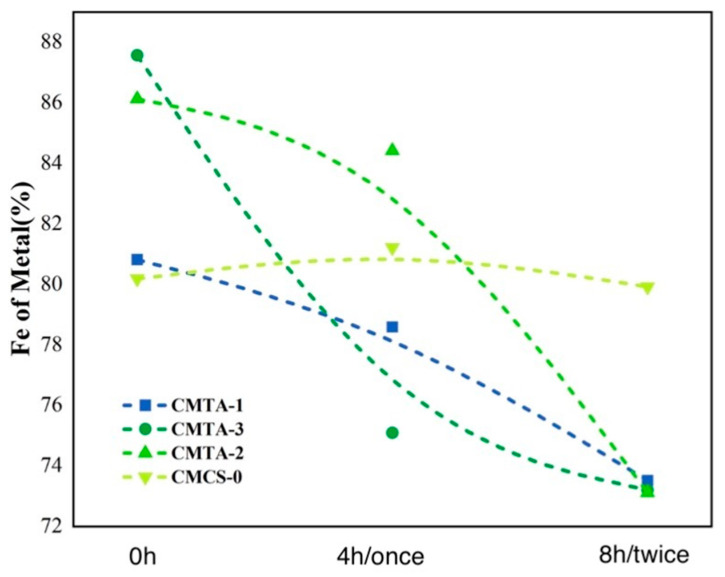
Changes in iron content of artefacts after cleaning and surface XRF testing.

For the characterisation of on-site cultural relic application effects, X-ray fluorescence (XRF) testing was used. Figure 10 shows that the iron content on the surface of cleaned cultural relic samples decreased significantly. According to Section 4.1.2., it takes 4 h to complete a single cleaning and peel off the film. After repeating the cleaning and peeling operation twice, the Fe content of the CMCS-0-treated area remained unchanged, while that of the CMTA-1-treated area decreased by 7.30%, that of the CMTA-2-treated area decrease 14.37%, and that of the CMTA-3-treated area decreased by 13.02%. CMTA-2 exhibited the highest removal efficiency with a stable decline rate. These results indicate that the CMCS/TA system can effectively clean and reduce the iron content in surface deposits on cultural relics.

#### 2.4.2. Colour Measurement

The a* value in the CIE Lab colour space was chosen as the core metric for quantitatively assessing the removal of ferruginous deposits in a chromatic aberrometry analysis. The a-axis reflects the red–green spectral variation (positive values are reddish, negative values are greenish), and ferruginous deposits shows reddish-brown colours in the visible light region. The CIE Lab system defines L as lightness [0 = black, 100 = white], a* as the red–green axis, and b* as the yellow–blue axis. The removal efficiency of rust deposits can be quantified by calculating the difference in a* before and after cleaning:

Δa*=before cleaning a*−after cleaning a*

**Figure 11:**
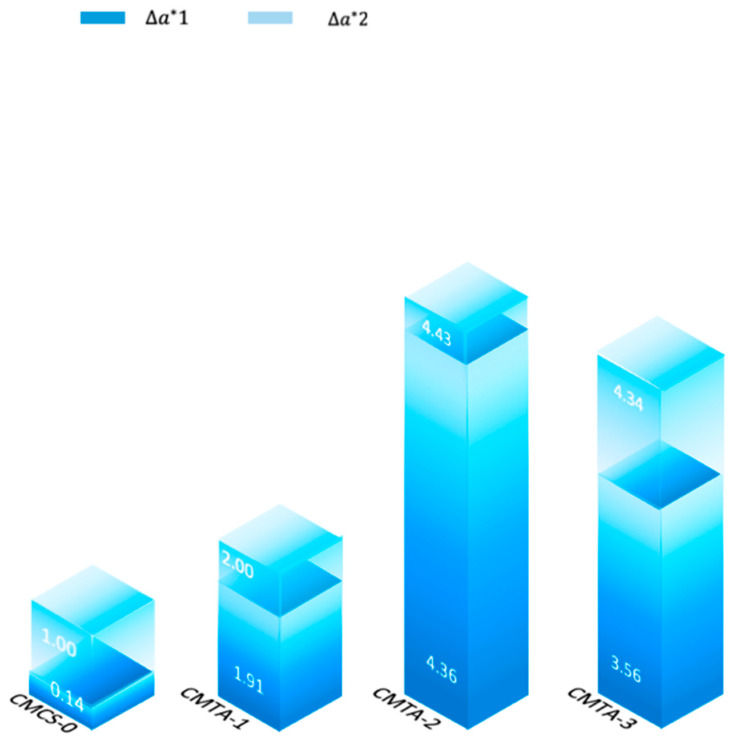
Difference between the colour of the artefacts after cleaning 1 and 2 times.

Figure 11 shows that Δa* is highly correlated with the change in Δ[Fe] (%), which indicates that it is highly efficient in ferruginous deposit removal through a chelation-adhesion mechanism. After the first time of cleaning, CMCS-0 (0.14) showed the smallest change, while CMTA-1 (1.91) and CMTA-3 (3.56) showed more obvious changes, and CMTA-2 (4.36) underwent the most significant change. This order was still maintained after the second time of cleaning. The colour difference indicated that the cleaning effect of the colloidal solution was remarkable and that additional cleaning can effectively enhance the efficiency (Table 4).

## 3. Conclusions

This study systematically investigated a composite colloidal system for cultural relic conservation. A laboratory analysis demonstrated that formulations achieving in situ gelation with optimal viscosity (260–300 mPa/s) facilitated the controllable removal of sedimentary layers (1 mm thickness) while operating within practical spraying parameters. The solution targets iron-rich phases, evidenced by a significant Fe/S atomic ratio reduction (Δ = –2.53) and minimised XRD high-intensity regions (19.90% at 1.325–1.400). Mechanistic studies confirmed the gel film functions through (1) TA-Fe coordination for chemical dissolution, (2) CMCS-PEG electrostatic attraction, and (3) CMCS carboxylate-Fe^3+^-accelerated reactions. The synergistic effect manifests as a substantial increase in roughness (ΔR = +0.37 mm), validating deposit adhesion during removal. Field validation on the Nanhai I shipwreck verified the system’s operational safety and efficacy, showing a 10–15% iron content reduction with obvious chromatic variation and clean post-treatment film separation. This provides a scientifically grounded solution for marine iron deposit conservation.

## 4. Materials and Methods

### 4.1. Materials

Iron (II) sulphide was purchased from Fuchen Chemical Reagent Co., Ltd. (Tianjin, China). Tannic acid was purchased from Sinopharm Chemical Reagent Co., Ltd. (Shanghai, China). Polyethylene glycol (PEG) (average molecular weight ≈ 2000) and carboxymethyl chitosan were purchased from Beijing Inokai Science and Technology Co., Ltd. (Beijing, China). Artefact samples were obtained from Zhongnanhai I Shipwreck Wooden Components from Guangdong Maritime Silk Road Museum (Yangjiang, Guangdong, China).

#### 4.1.1. Sample Preparation

Firstly, 5 g of FeS powder was compressed at 15 Mpa for 30 s to form a 1 × 1 cm sample, which was then placed in a high-temperature furnace and heated up to 300 °C at a rate of 5 °C/min and held for 1 h for firing. After the samples were completely cooled, a 10% concentration of aqueous PEG solution was added dropwise to completely wet the samples, which were reinforced and dried naturally, and this step was repeated twice to simulate the current state of the surface deposits on wooden hulls, and the prepared simulated samples did not disintegrate after being immersed in the water for 24 h.

#### 4.1.2. Preparation of the Gel Cleaning Solution

Cleaning gels with different concentrations of CMCS/TA were prepared and tested. Firstly, an appropriate amount of carboxymethyl chitosan was dissolved in deionised water and stirred for 20 min until the solution was completely transparent. After that, an appropriate amount of tannic acid was also dissolved in deionised water, and the tannic acid solution was added to the carboxymethyl chitosan solution to trigger coordinated gelation via thorough mixing. The resulting sol–gel transition solution was transferred to a 30 mL spray bottle fitted with a nozzle of 2 mm inner diameter. The solution was then evenly applied to the sample surface via spraying. Finally, the sample was air-dried at room temperature under ambient humidity with light protection. Upon complete drying, a coherent hydrogel film formed. Partial spontaneous delamination of the film layer occurred, which could be completely removed using tweezers (Table 5).

### 4.2. Field Applications for Heritage Conservation

**Figure 12:**
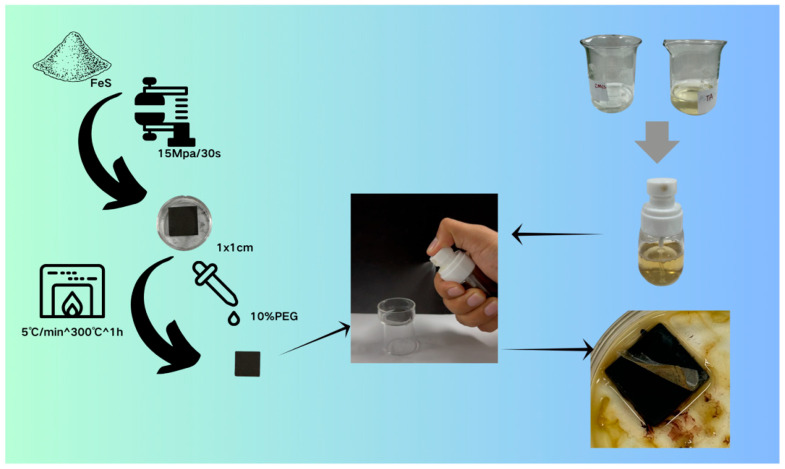
Overall process flow diagram.

**Figure 13:**
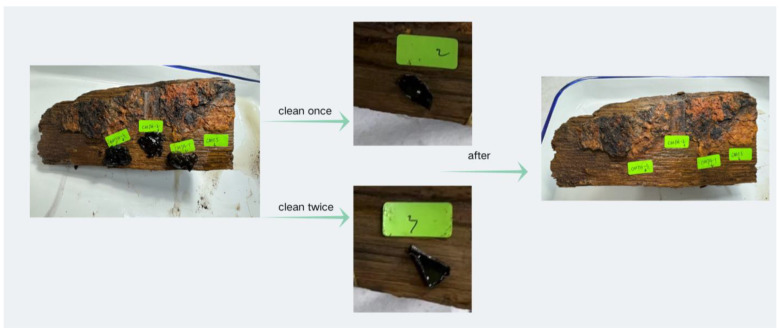
Flow chart for cleaning of wood blocks of Nanhai I shipwreck.

The Guangdong Provincial Maritime Silk Road Museum, home to the ‘Nanhai I’ out-of-the-water cultural relic protection site, was chosen for the implementation of in situ experiments, selecting hull samples with typical corrosion characteristics (ferruginous deposit attachment) as the object of study. Four treatment groups were set up: CMTA-1, CMTA-2, CMTA-3 (tannic acid/carboxymethyl chitosan composite colloid solution gradient concentration group), and CMCS-0 (blank control group). Figure 12 and Figure 13 show two repetitive laminating/peeling test procedures (120 ± 5 min intervals) implemented at room temperature (23 ± 2 °C) environment using standardised procedures.

### 4.3. Characterisation Methods

#### 4.3.1. Viscosity

Use an NDJ-5S digital display rotational viscometer, with a rotor specification of No. 0/No. 1 (Puyun, Shenzhen, China), and a speed setting interval of 6–30 rpm. Use a horizontal adjustment screw to make the horizontal bubble centred, respectively; after the configuration of the sample poured into the viscometer, the rotor is completely submerged in the liquid and remains stable, to be displayed at the tensor angle stabilised at 50%; record the viscosity value at this time.

#### 4.3.2. Fourier Transform Infrared Spectrometer (FTIR)

A Thermo Fisher Nicolet iS 5 Fourier Transform Infrared Spectrometer equipped with a DTGS detector (Thermo Fisher Scientific, Waltham, MA, USA) was used to characterise the raw material and the cleaning solution, and to analyse the cross-linking mechanism of the solution to form a film as well as the cleaning mechanism. FTIR analysis was performed on the cleaned film samples. The stripped cleaned film was mixed with KBr powder by grinding and then compressed in a press mould for 1 min. the FTIR spectrophotometer was used in the range of 500–4000 cm^−1^ with a resolution of 16 scans.

Two-dimensional trace FTIR mapping was used to confirm the residue on the surface of the cleaned samples, as well as the membrane cleaning efficiency. A Thermo Fisher Nicolet in 10 (Thermo Fisher Scientific, Waltham, MA, USA) was used, equipped with a microscope for microanalysis. A liquid nitrogen cooled MCT detector was used to capture signals in the 4000–650 cm^−1^ range with a Fourier Transform Infrared (FTIR) spectral resolution of 4 cm^−1^ and 128 scans.

#### 4.3.3. Three-Dimensional Super Depth-of-Field Microscope

Microscopic images of the demarcation line before and after the cleaning of the simulated samples were taken using a 3D Super Depth-of-Field Microscope VHX-S660E (Keyence, Minato, Osaka, Japan). The lens used was a VH-ZST with a magnification of 30, and the simulated samples were scanned in 3D to test the sample roughness.

#### 4.3.4. Scanning Electron Microscope (SEM)

Scanning electron microscope (SEM) images of the surface of the simulated samples before and after cleaning as well as before and after film cleaning were acquired using a Hitachi Regulus 8100 cold-field SEM (Hitachi, Tokyo, Japan). All samples were coated with a gold sputtering coating, and microscopic morphology was observed at an accelerating voltage of 10/15 KV and a working distance of 15 mm.

#### 4.3.5. X-Ray Fluorescence Spectroscopy

A hand-held X-ray fluorescence spectrometer (model: S1 TITAN 500; detection method: MultiMatrix4015, time: 40 s) (Bruker, Billerica, MA, USA)was used to perform a semi-quantitative analysis of the surface elements, and to obtain data on the changes in the content of Fe/S and other characteristic elements before and after treatment.

#### 4.3.6. Spectrophotometric Colourimeter

A portable spectrophotometric colorimeter (model: 3nh ST60) (3nh, Shenzhen, China) system configured with a D/8 measuring structure was used to measure Lab* chromaticity spatial parameters and calculate Δa values to assess changes in surface colour before and after cleaning.

## Figures and Tables

**Table 1:** Microscope analysis results of each sample.

	Thickness Removal (mm)	Roughness Change (mm) (ΔR = Before-After)
CMCS	0.49	+0.02
CMTA-1	1.02	+0.15
CMTA-2	1.01	+0.37
CMTA-3	0.82	+0.17
TA	0.79	−0.20

**Table 2:** EDS results of each sample.

Number/Mass (%)			C	O	Fe	S	Fe/S Atomic Ratio
CMCS	1	uncleaned	4.00	1.97	17.31	9.41	1.84
	2	cleaned	1.93	2.99	44.46	20.54	2.16
CMTA-1	1	uncleaned	8.08	7.22	17.23	2.02	8.53
	2	cleaned	3.28	5.81	32.31	5.70	5.67
CMTA-2	1	uncleaned	6.37	6.77	19.00	3.46	5.49
	2	cleaned	2.61	6.43	29.93	10.10	2.96
CMTA-3	1	uncleaned	7.31	6.28	8.67	1.68	5.16
	2	cleaned	2.95	3.47	23.44	8.72	2.69
TA	1	uncleaned	10.00	7.79	19.45	6.13	3.17
	2	cleaned	4.87	7.55	34.26	12.78	2.68

**Table 3:** Percentage of area of each sample in different intensity ranges under the 850 cm^−1^ peak of the FT-IR measurements.

	CMCS	CMTA-1	CMTA-2	CMTA-3	TA
1.100–1.175	0.4%	0%	19.99%	17.13%	0%
1.175–1.325	48.19%	56.44%	60.11%	79.86%	71.79%
1.325–1.400	51.41%	43.56%	19.90%	3.01%	28.21%

**Table 4:** The colour of artefacts after cleaning.

Sample		Uncleaning	Once	Twice
CMCS-0	L *	31.11	30.95	30.09
	a *	7	7.14	6
	b *	14.68	14.97	11.1
CMTA-1	L *	31.23	30.2	31.94
	a *	6.8	4.89	4.8
	b *	14.1	11.04	10.71
CMTA-2	L *	30.11	30.98	32.19
	a *	8.16	3.8	3.73
	b *	15.01	10.3	10.03
CMTA-3	L *	30.44	30.95	31.3
	a *	8.51	4.95	4.17
	b *	15.39	11.51	11.03

* Indicate the difference between the inspected sample and the standard sample.

**Table 5:** Composition of CMCS/TA cleaning solution.

Code	CMCS/H_2_O (g/mL)	TA/H_2_O (g/mL)
CMCS	0.2/10	/
CMTA-1	0.2/10	0.05/5
CMTA-2	0.4/10	0.05/5
CMTA-3	0.6/10	0.05/5
TA	/	0.15/15

## Data Availability

The original contributions presented in this study are included in the article/Supplementary Materials. Further inquiries can be directed to the corresponding authors.

## Supplementary Materials

The following supporting information can be downloaded at: https://www.mdpi.com/article/10.3390/gels11070543/s1, Figure S1: SEM images of gel film: (a) CMCS, (b) CMTA-1, (c) CMTA-2, (d) CMTA-3.

## References

[1] Han Y., Du J., Huang X., Ma K., Wang Y., Guo P., Li N., Zhang Z., Pan J. (2021). Chemical Properties and Microbial Analysis of Waterlogged Archaeological Wood from the Nanhai No. 1 Shipwreck. Forests.

[2] Kangfan W., Tianshe Z. The Underwater Archaeological Career Progress of Research in China Showed by “Nanhai I”. Proceedings of the 2nd International Conference on Contemporary Education, Social Sciences and Ecological Studies (CESSES 2019).

[3] Peining L. (2020). The trade patterns of the South China Sea during the Song period. Asian Archaeol..

[4] Jiao P., Yeqing H., Cen W., Jing D., Yu W., Yue C., Xinduo H., Kaixuan M., Zhiguo Z., Naisheng L. (2023). Analysis of microbial community and biodeterioration of maritime cultural relics (ironware, porcelain, axes, hull wood) from the Nanhai No. 1 shipwreck. Ann. Microbiol..

[5] Ke-jia H., Jing D., Jian Z., Nai-sheng L., Yue C., Yuan-yuan W. (2021). Mapping Analysis by mu-X-Ray Fluorescence for Waterlogged Archaeological Wood From” Nanhai No. 1” Shipwreck. Spectrosc. Spectr. Anal..

[6] Hongying Z., Dawa S., Zhiguo Z., Qinglin M. (2022). Characterization of degradation and iron deposits of the wood of Nanhai I shipwreck. Herit. Sci..

[7] Mathilde M., Magdalena A.-B., Charlène P., Emilie C., Elodie G., Céline R., Edith J. (2020). Characterization of model samples simulating degradation processes induced by iron and sulfur species on waterlogged wood. Microchem. J..

[8] Magdalena B., Callum A.S.H. (2021). Conservation of Waterlogged Wood—Past, Present and Future Perspectives. Forests.

[9] Aslı Gökçe K., Namık K., Donna C.A. (2023). Analyses of Sulfur and Iron in Waterlogged Archaeological Wood: The Case of Polyethylene-Glycol-Treated Yenikapı 12 Shipwreck. Forests.

[10] Shen D. (2020). Iron Sulfide of Marine Archaeological Wood.

[11] Liu Z., Fu T., Hu C., Shen D., Macchioni N., Sozzi L., Chen Y., Liu J., Tian X., Ge Q. (2018). Microbial community analysis and biodeterioration of waterlogged archaeological wood from the Nanhai No. 1 shipwreck during storage. Sci. Rep..

[12] Rémazeilles C., Tran K., Guilminot E., Conforto E., Refait P. (2013). Study of Fe (II) sulphides in waterlogged archaeological wood. Stud. Conserv..

[13] Xunming G., Jian Z., Jiahui L., Lihua F., Dong Z. (2024). How do water and acid in marine archaeological wood affect its mechanical properties?. J. Cult. Herit..

[14] Caire J., Bouh A., Guilminot E. Numerical modelling of electrophoresis applied to restoration of archaeological organic materials. Proceedings of the COMSOL Conference.

[15] Monachon M., Pelé-Meziani C., Ganesan S., De Weck S., Moll-Dau F., Schramm J., Schmidt-Ott K., Joseph E. (2023). Assessing the versatility of bioextraction to preserve waterlogged wood. Forests.

[16] Yishu W., Zijun Z., Jianqun L., Qinglin M., Linxu C. (2024). A new bio-oxidation method for removing iron deposits from waterlogged wood of Nanhai I shipwreck, Guangdong, China. Eng. Microbiol..

[17] Céline R., Laure M., François L., Nicolas P., Egle C., Marine C., Philippe R., Loïc C. (2019). Post-treatment Study of Iron/Sulfur-containing Compounds in the Wreck of Lyon Saint-Georges 4 (Second Century ACE). Stud. Conserv..

[18] Almkvist G., Persson I. (2006). Extraction of iron compounds from wood from the Vasa. Holzforschung.

[19] Zhiguo Z., Naisheng L., Xingling T., Jie L., Dawa S. (2014). Research on the removal of the iron sulfides in the Qing Dynasty marine shipwreck, Ningbo Xiaobaijiao No. 1. Sci. Conserv. Archaeol..

[20] Eric S., Robert B., Held B., Jurgens J., Cook D., Drews M., Hand S., Betty S. (2005). An Evaluation of Supercritical Drying and PEG/Freeze Drying of Waterlogged Archaeological Wood.

[21] Lindfors E.-L., Lindström M., Iversen T. (2008). Polysaccharide degradation in waterlogged oak wood from the ancient warship Vasa. Holzforschung.

[22] Pecoraro E., Pelé-Meziani C., Macchioni N., Lemoine G., Guilminot E., Shen D., Pizzo B. (2023). The removal of iron from waterlogged archaeological wood: Efficacy and effects on the room temperature wood properties. Wood Mater. Sci. Eng..

[23] Rapti S., Rivers S., Pournou A. Removing iron corrosion products from museum artefacts: Investigating the effectiveness of innovative green chelators. Proceedings of the SCinTE 2015.

[24] Pecoraro E., Pelé-Meziani C., Macchioni N., Lemoine G., Guilminot E., Pizzo B. (2022). Effects of iron removal treatments on the chemical and viscoelastic properties of waterlogged wood. J. Cult. Herit..

[25] Yang Y., Lian X., Yang Z., Zhou Y., Zhang X., Wang Y. (2021). Self-shaping microemulsion gels for cultural relic cleaning. Langmuir.

[26] Upadhyaya L., Singh J., Agarwal V., Tewari R.P. (2013). Biomedical applications of carboxymethyl chitosans. Carbohydr. Polym..

[27] Li X., Chen S., Zhang B., Li M., Diao K., Zhang Z., Li J., Xu Y., Wang X., Chen H. (2012). In situ injectable nano-composite hydrogel composed of curcumin, N, O-carboxymethyl chitosan and oxidized alginate for wound healing application. Int. J. Pharm..

[28] Liefeng H., Panpan Z., Xin W., Xu C., Jiejie Q., Rupei T. (2017). pH-sensitive carboxymethyl chitosan hydrogels via acid-labile ortho ester linkage for potential biomedical applications. Carbohydr. Polym..

[29] Andrew B., Brian W.B. (2022). Biomedical applications of tannic acid. J. Biomater. Appl..

[30] George K.B.L., Herbert M.S., Marcelo H.-L. (1999). Polyphenol tannic acid inhibits hydroxyl radical formation from Fenton reaction by complexing ferrous ions. Biochim. Biophys. Acta (BBA)—Gen. Subj..

[31] Harmon B.A., Ahmad B.R., Brushmiller J.G. (1994). Photochemical and spectroscopic studies of complexes, of iron(III) with citric acid and other carboxylic acids. Inorganica Chim. Acta.

[32] Tang L., Gong J., Li J., Fang S., Wang Y., Zhou H. (2023). Synergistic effect between tannic acid-Fe3+ and chitosan hydrogel-coated covalent organic framework: Endowing better nanofiltration performance and stability. Sep. Purif. Technol..

[33] Üçer A., Uyanik A., Aygün Ş. (2006). Adsorption of Cu (II), Cd (II), Zn (II), Mn (II) and Fe (III) ions by tannic acid immobilised activated carbon. Sep. Purif. Technol..

[34] Zhang J., Fu C., Tian T., Batur S., Lv J., Xie Q., Kong L., Yang C., Zhang Z. (2025). In Situ Ultrafast Self-gelling Coacervate Powder with Antibacterial, Antioxidant, and Robust Wet Adhesion Properties for Hemostasis and Wound Healing. Adv. Funct. Mater..

[35] Wu Z.-c., Tao T.-x., Wang X.-Q. (2004). The IR spectra of complexes of fiber containing amidoxime groups with Fe (III), Co (II), Ni (II), Cd (II) and Hg (II). Guang Pu Xue Yu Guang Pu Fen Xi = Guang Pu.

[36] Lin Q.-q., Huang Y.-x., Zhang Y.-h., Rao F., Yu W.-j. (2021). Measurement of surface roughness of Salix psammophila scrimber using stylus profilometer and extended depth-of-field 3D microscope. J. For. Eng..

[37] Bhargava R., Wall B.G., Koenig J.L. (2000). Comparison of the FT-IR Mapping and Imaging Techniques Applied to Polymeric Systems. Appl. Spectrosc..

[38] Jeong Y.-I., Jin S.-G., Kim I.-Y., Pei J., Wen M., Jung T.-Y., Moon K.-S., Jung S. (2010). Doxorubicin-incorporated nanoparticles composed of poly (ethylene glycol)-grafted carboxymethyl chitosan and antitumor activity against glioma cells in vitro. Colloids Surf. B Biointerfaces.

