# Supplementary figures and images for: In Situ Gel-Forming System for the Removal of Ferruginous Deposits on Nanhai I Shipwreck
Source: Gels. 2025 Jul 12;11(7):543. doi: 10.3390/gels11070543 (PMC12295655; doi:10.3390/gels11070543)

Supplementary Materials:

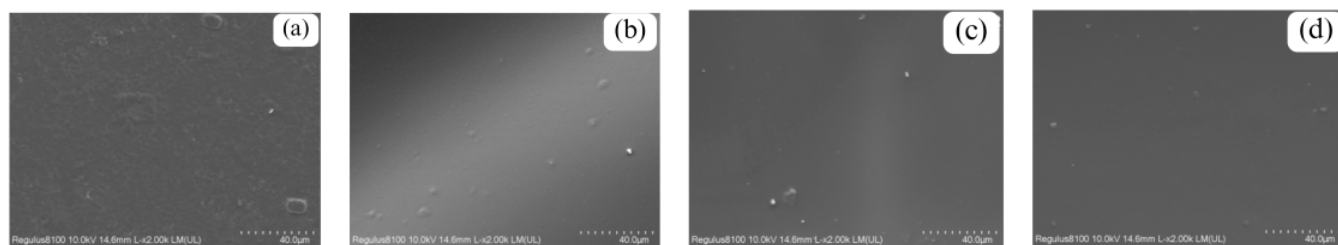

**Figure S1.** SEM images of gel film (a) CMCS, (b) CMTA-1, (c) CMTA-2, (d) CMTA-3.

Supplement: Supplementary file 1 [file gels-11-00543-s001.zip › gels-3713557-supplementary.pdf]
